# The mitotic spindle: linking teratogenic effects of Zika virus with human genetics?

**DOI:** 10.1186/s13039-016-0240-1

**Published:** 2016-04-19

**Authors:** Joern Bullerdiek, Andreas Dotzauer, Ingrid Bauer

**Affiliations:** Centre for Human Genetics, University of Bremen, Leobener Str. ZHG, D-28359 Bremen, Germany; Institute of Medical Genetics, University Rostock Medical Center, Ernst-Heydemann-Strasse 8, D-18057 Rostock, Germany; Laboratory of Virus Research, University of Bremen, Leobener Straße/UFT, 28359, D-28359 Bremen, Germany

**Keywords:** Zika virus, Microcephaly, Chorioretinopathy, Mechanisms, Syndromes, Genetics, Phenocopy

## Abstract

**Background:**

Recently, an association between Zika virus infection and microcephaly/ocular findings was found to be reasonable e.g. because of the demonstration that the virus was found in the brain of a fetus after presumed maternal infection. Although there is no proof yet for a causal relationship, for an appropriate risk calculation efforts are urgently needed to either establish or disprove this assumption.

**Presentation of the hypothesis:**

On the basis of inherited syndromes combining microcephaly with ocular findings similar to those associated with Zika infections, we have hypothesized that the impairment of the proper function of the mitotic apparatus is a possible mechanism by which Zika can exert teratogenic effects.

**Testing the hypothesis:**

A bundle of well-known cytogenetic and molecular-cytogenetic methods (e.g. formation of micronuclei, chromosomal lagging, immunofluorescence of centrosomes) to evaluate proper function, maintenance, and establishment of the mitotic spindle poles can be applied on infected cells. Also, the viral proteins can be tested for their possible interaction with proteins encoded by genes involved in inherited syndromes with microcephaly and ocular findings resembling those in presumed cases of intrauterine ZIKV infection.

**Implications of the hypothesis:**

Once proved, this hypothesis allows for a targeted approach into mechanisms of possible relevance as e.g. if different strains of the virus are implicated in the teratogenic effects to the same or a different extent.

## Background

The Zika virus (ZIKV) is an arbovirus of the flaviviridae family with a single-stranded RNA genome of approximately 10,000 nucleotides (GenBank: KU647676.1, GenBank: KU509998.1, GenBank: KU501215.1). It is vertically transmitted by mosquitoes of the *Aedes* family (*Aedes spec.*). Accordingly, spreading of the virus will coincide primarily with the geographical distribution of mosquitoes of this family as e.g. *Aedes aegypti*. On the other hand, some recent cases of *ZIKV* infections suggest sexual transmission of the virus by semen as well.

Quite recently, in Brazil microcephaly [1] and chorioretinopathy [2, 3] as well as signs of *in utero* growth restriction [4] were found to be epidemiologically associated with either clinically suspected maternal ZIKV infections during pregnancy and/or positive qualitative RT-PCR tests for ZIKV in maternal blood, urine or both. The association between ZIKV infection and microcephaly was deduced also from a recent more detailled case report: In a pregnant women who has had a febrile illness with rash at the end of the first trimester of her pregnancy, ultrasonography at the 29th gestational week showed microcephaly with calcifications in the fetal brain and placenta. After termination of the pregnancy, the presence of ZIKV was demonstrated in the fetal brain and the whole genome of ZIKV was recovered by using next generation sequencing [4], (GenBank deposition number KU527068). A causal relationship has been presumed [5, 6] and this association was the major reason that prompted the WHO to declare a Public Health Emergency of International Concern (PHEIC). While public health authorities advised pregnant travellers to “consider avoiding travel to an area where active Zika transmission is being reported“(see: https://www.gov.uk/guidance/zika-virus), proof for a causal relationship between the infection and the neurotopic teratogenic effects has not been established yet and possible underlying mechanisms remain to be elucidated.

An interesting aspect of the nearly endemic occurrence of microcephaly in Brazil is its coincidence with ocular abnormalities. As reported in a study from Salvador, Brazil, out of twenty-nine infants diagnosed with microcephaly having a presumed diagnosis of ZIKV infection of their mothers based on clinical signs during pregnancy, ten presented with ocular abnormalities that were bilateral in seven cases. Mostly they revealed focal pigment mottling of the retina and chorioretinal atrophy (11/17 eyes) and optic nerve abnormalities (8/17 eyes) [7]. Similar findings are reported in a study from Recife, Brazil [2].

## Hypothesis generation

In order to understand the possible causal relationship between the viral infection and microcephaly, we looked for genetic disorders showing the same coincidence of microcephaly with ocular abnormalities as reported from Brazil. As to severe congenital microcephaly in general, a large number of cases can be correlated with inherited gene mutations. Of these, those causing mitotic dysfunctions can be assumed to play a major role in the development of microcephaly. In addition to microcephaly associated with other syndromic features, most of the genetic causes of ‘primary’ developmental microcephaly are known to be associated with dysfunction of centrosomal proteins controlling the mitotic spindle assuring normal cell proliferation during mitosis [8]. Akin to isolated microcephaly, the combination of microcephaly with chorioretinal abnormalities also seems to be related to mutations of genes involved in proper spindle function:

As for autosomal-dominant inheritance, a phenotypic overlap can be noted between two syndromes i.e. MLCRD (microcephaly, primary lymphedema, and chorioretinal dysplasia) syndrome (MIM 152950, OMIM) and CDMMR (chorioretinal dysplasia, microcephaly, and mental retardation) syndrome (MIM 156590) [9, 10]. In MLCRD the main ocular abnormalities are bilateral chorioretinopathy, but optical nerve abnormalities do occur as well [11]. Interestingly, in both syndromes heterozygous mutations of the *KIF11* (*Kinesin Family Member 11*) gene encoding EG5, a spindle motor protein of the kinesin-5 family were found [10]. One possible explanation for families with either MLCRD or CDMMR where no variants in the coding region of *KIF11* were detected [10] is that they represent phenocopies of the syndromes. While Eg5 is essential for the organization and maintenance of mitotic and meiotic spindles in dividing cells [12, 13] by acting during poleward movement [14] Ferhat et al. [15] were able to demonstrate the relevance of EG5 for neuronal development in postmitotic cells as well. Remarkably, viral proteins are able to interact with EG5. Liu et al. [16] recently demonstrated that Tat, the transactivation factor of human immunodeficiency virus type 1 interacts with Eg5 by allosterically modulating the ATPase activity of Eg5.

Autosomal recessive forms of microcephaly with chorioretinopathy are caused by mutations of the genes of the master regulator of centriole duplication, the PLK4 kinase (MCCRP2, MIM 616171) [17], and their substrates TUBGCP4 (MCCRP3, MIM 616335) [18] and TUBGCP6 (MCCRP1, MIM 251270) [17]. As essential components of the spindle formation, all three proteins are also involved in the proper mitotic function.

Considering possible ZIKV-related birth defects as phenocopies of the syndromes described above and taking into account that viruses are able to interfere with the mitotic spindle [16, 19, 20], we would like to advance the hypothesis that ZIKV, either directly or indirectly, exerts its teratogenic effects by interacting with proteins engaged in the assembly of the mitotic apparatus. The hypothesis is supported by the findings that cytopathogenic effects of ZIKV infections in cell culture seem to depend on the cell line used, that ZIKV is able to infect neural stem cells, hampering cell growth and interrupting the cell division cycle, and that viral proteins could be detected in the nucleus of infected cells [21–23].

## Testing the hypothesis

To test this hypothesis, well-known cytogenetic and molecular-cytogenetic methods to evaluate function, maintenance and establishment of the mitotic spindle poles can be applied on infected cells. E.g. they can be tested for the formation of micronuclei, chromosomal lagging, and the number of centrosomes. Also, proteins encoded by genes involved in inherited syndromes with microcephaly and occular findings resembling those in presumed cases of intrauterine ZIKV infection should be tested for possible interaction with viral proteins.

Vice versa, as a straightforward approach one might also consider testing newborns with microcephaly that may be associated with maternal ZIKV infection for germline mutations of the genes mentioned but the rarity of these syndromes makes such an explanation unlikely.

## Implications of the hypothesis

With regard to ZIKV infection, there is a considerable lack of knowledge on basic mechanisms of the virus’ interaction with host cells. Accordingly, it may take months or even years to test whether or not ZIKV infection has a teratogenic potential. We have proposed a hypothesis that allows for a targeted approach into mechanisms of possible relevance.

## Abbreviations

CDMMR: chorioretinal dysplasia, microcephaly, and mental retardation syndrome
EG5: alternative abbreviation of the gene product of *KIF11*
*KIF11*: Kinesin Family Member 11 gene, alternative title of the gene product is EG5
MCCRP1: Microcephaly and Chorioretinopathy, Autosomal Recessive, 1
MCCRP2: Microcephaly and Chorioretinopathy, Autosomal Recessive, 2
MCCRP3: Microcephaly and Chorioretinopathy, Autosomal Recessive, 3
MLCRD: microcephaly, primary lymphedema, and chorioretinal dysplasia syndrome
OMIM: Online Mendelian Inheritance in man (http://www.omim.org/)
PLK4: Polo-like Kinase 4
RT-PCR: reverse transcriptase polymerase chain reaction
TUBGCP4: Tubulin-Gamma Complex-Associated Protein 4
TUBGCP6: Tubulin-Gamma Complex-Associated Protein 6
WHO: World Health Organisation
ZIKV: Zika virus
